# Dynamic Contrast-Enhanced MR Imaging Predicts Local Control in Oropharyngeal or Hypopharyngeal Squamous Cell Carcinoma Treated with Chemoradiotherapy

**DOI:** 10.1371/journal.pone.0072230

**Published:** 2013-08-07

**Authors:** Shu-Hang Ng, Chien-Yu Lin, Sheng-Chieh Chan, Tzu-Chen Yen, Chun-Ta Liao, Joseph Tung-Chieh Chang, Sheung-Fat Ko, Hung-Ming Wang, Shiang-Fu Huang, Yu-Chun Lin, Jiun-Jie Wang

**Affiliations:** 1 Molecular Imaging Center, Chang Gung Memorial Hospital, Chang Gung University, Kueishan,, Taoyuan, Taiwan; 2 Department of Diagnostic Radiology, Chang Gung Memorial Hospital, Chang Gung University, Kueishan,, Taoyuan, Taiwan; 3 Department of Medical Imaging and Radiological Sciences, Chang Gung Memorial Hospital, Chang Gung University, Kueishan,, Taoyuan, Taiwan; 4 Department of Radiation Oncology, Chang Gung Memorial Hospital, Chang Gung University, Kueishan,, Taoyuan, Taiwan; 5 Department of Nuclear Medicine, Chang Gung Memorial Hospital, Chang Gung University, Kueishan,, Taoyuan, Taiwan; 6 Department of Otorhinolaryngology, Head and Neck Surgery, Chang Gung Memorial Hospital, Chang Gung University, Kueishan,, Taoyuan, Taiwan; 7 Department of Medical Oncology, Chang Gung Memorial Hospital, Chang Gung University, Kueishan,, Taoyuan, Taiwan; Northwestern University Feinberg School of Medicine, United States of America

## Abstract

The role of pretreatment dynamic contrast-enhanced perfusion MR imaging (DCE-PWI) and diffusion-weighted MR imaging (DWI) in predicting the treatment response of oropharyngeal or hypopharyngeal squamous cell carcinoma (OHSCC) to chemoradiation remains unclear. We prospectively investigated the ability of pharmacokinetic parameters derived from pretreatment DCE-PWI and DWI to predict the local control of OHSCC patients treated with chemoradiation. Between August, 2010 and March, 2012, patients with untreated OHSCC scheduled for chemoradiation were eligible for this prospective study. DCE-PWI and DWI were performed in addition to conventional MRI. The relationship of local control with the following clinical and imaging variables was analyzed: the hemoglobin level, T-stage, tumor location, gross tumor volume, maximum standardized uptake value, metabolic tumor volume and total lesion glycolysis on FDG PET/CT, transfer constant (*K* ^*trans*^), volume of blood plasma and volume of extracellular extravascular space on DCE-PWI, and apparent diffusion coefficient on DWI of the primary tumor. The patients were also divided into a local control group and a local failure group, and their clinical and imaging parameters were compared. There were 58 patients (29 with oropharynx squamous cell carcinoma [SCC] and 29 with hypopharynx SCC) with successful pretreatment DCE-PWI and DWI available for analysis. After a median follow-up of 18.2 months, 17 (29.3%) participants had local failure, whereas the remaining 41 patients achieved local control. Univariate analysis revealed that only the *K* ^*trans*^ value was significantly associated with local control (*P* = 0.03). When the local control and local failure groups were compared, significant differences were observed in *K* ^*trans*^ and the tumor location (*P* = 0.01 and *P* = 0.04, respectively). In the multivariable analysis, only *K* ^*trans*^ was statistically significant (*P* = 0.04). Our results suggest that pretreatment *K* ^*trans*^ may help predict the local control in OHSCC patients treated with chemoradiation.

## Introduction

Oropharyngeal and hypopharyngeal squamous cell carcinomas (OHSCC) are common malignant tumors of the head and neck that has long been considered as having similar risk factors and lymphatic drainage. However, human papillomavirus (HPV) is recently recognized to play a role in the pathogenesis of a subset of clinically and molecularly distinct head and neck squamous cell carcinomas (HNSCC), most commonly located in the oropharynx. Patients with HPV-positive oropharyngeal SCC typically have lower age at onset, limited tobacco exposure, and more favorable prognosis compared with their HPV-negative counterparts [1,2]. Currently, an organ-preservation approach with chemoradiation has become an accepted treatment option for OHSCC [3]. However, such treatment mode is not always successful, and the reported local control rates were about 80% [4,5]. An early prediction of failure may allow for therapeutic modification, including the selection of suitable candidates for surgery in resectable OHSCC and the intensification of chemoradiation or the use of immunotherapy in unresectable cases [6,7]. Imaging biomarkers are being developed to identify tumors that are non-responsive to chemoradiation.

With the advance of MRI technology, diffusion-weighted MR imaging (DWI) and dynamic contrast-enhanced perfusion MR imaging (DCE-PWI) have become potential tools to evaluate the functional aspects of HNSCC. DWI is a quick MRI technique that can quantify the diffusion of water molecules in tissues using apparent diffusion coefficient (ADC). ADC has an inverse correlation with cell density [8,9], and the highly solid tumors (characterized by a lower ADC) may respond better to chemoradiation than tumors with a higher ADC. Because DWI takes only a few minutes to be acquired, it can be easily incorporated into routine head and neck MRI protocols. DCE-PWI is a functional MRI technique that based on sequential imaging obtained during the passage of a contrast agent through the tissue of interest. It is capable of probing the microvascular environment in the lesion, such as perfusion, the permeability of blood vessels and the volume of the extracellular space [10,11]. Since tumor oxygenation and tumor microvascular attenuation could be related to radiosensitivity [12], DCE-PWI is expected to help predict tumor responses to chemoradiation.

To date, only limited data are available regarding the use of pretreatment DCE-PWI or DWI to predict the chemoradiation response of HNSCC, and conflicting results have been reported [4,7,13–18]. The hemoglobin (Hb) level [4,19,20], T-stage [21], tumor volume [4,5,22–24], as well as fluorodeoxyglucose-positron emission tomography (FDG-PET)/CT parameters, including the maximum standardized uptake value (SUV_max_), metabolic tumor volume (MTV), and total lesion glycolysis (TLG) [25–27] have been reported to be correlated with local control, but reliable prediction of treatment response remains difficult. Therefore, we conducted a prospective study to determine whether the pretreatment DWI, DCE-PWI and the other reported potential factors can offer predictive power regarding to the local control of OHSCC in patients treated with definitive chemoradiation. To our knowledge, this is the first prospective study about the local control of OHSCC in patients undergoing chemoradiation evaluated with DWI and DCE-PWI, as well as FDG PET/CT.

## Patients and Methods

### Ethics Statement

The Institutional review board of the Chang Gung Memorial Hospital approved the study protocol (protocol no. 98-3582B) in September 2009. All participants gave written informed consent and the study followed the Declaration of Helsinki.

### OHSCC Patients

The study protocol was approved by the local Institutional Review Board and written informed consent was obtained from all participants. Patients with newly diagnosed OHSCC scheduled for chemoradiation with curative intent were eligible for this prospective study. The study subjects underwent a thorough pretreatment evaluation, including MRI and ^18^F-FDG PET/CT. The exclusion criteria included the presence of a previous head or neck malignant tumor, a second malignant tumor or distant metastases, renal failure, or other contraindications for MRI. All of the patients were treated with intensity-modulated radiotherapy with a 6-MV X-ray at 2 Grays (Gy) per fraction, with five fractions per week. The radiotherapy dose was 46-50 Gy for all subclinical risk areas, including the neck lymphatics, and 72 Gy for primary tumor and grossly involved nodal disease. Concurrent chemotherapy consisted of cisplatin 50mg/m^2^ on day 1, oral tegafur 800 mg/day plus leucovorin 60 mg/day from day 1 to day 14. It was repeated every two weeks through the radiotherapy course [28].

Following treatment, the patients underwent a routine follow-up clinical examination that included a physical examination and fiberoptic pharyngoscopy every 1 to 3 months. A follow-up MRI was performed 3 months after the treatment was completed, and additional MRI or CT was performed every 6 months thereafter or on clinical deterioration. Endoscopic biopsy, ultrasonographic guided fine needle aspiration, or CT-guided biopsy was performed for any suspicious residual/recurrent tumors, if possible. If biopsy of the lesion of interest was not feasible or yielded a negative result, close clinical and imaging follow-up was pursued. Those patients without pathologically proven recurrence were followed up for at least 12 months after treatment or until death.

### MRI with DWI and DCE-PWI

The MR imaging study was performed using a 3 Tesla MR scanner (Magnetom Trio with TIM, Siemens, Erlangen, Germany). Conventional MRI of the head and neck region was obtained in the axial and coronal projections with T2-weighted turbo spin echo (TSE) sequence with fat saturation plus T1-weighted TSE sequence, as well as with postcontrast fat-saturated T1-weighted TSE sequence.

The DWI was acquired using a single shot spin-echo echo-planar imaging with modified Stejskal-Tanner diffusion gradient pulsing scheme. Motion-probing gradients with a b-value of 800 s/mm^2^ were applied along three orthogonal directions. The imaging slice and coverage were identical to the T1 and T2 weighted images. The repetition time (TR) and echo time (TE) were 8,200 ms and 84 ms, respectively.

DCE- PWI was acquired by using a 3D T1-weighted spoiled gradient-echo sequence with the following parameters: TR/TE = 3.5/1.13 ms, 230×230-mm field of view, 108×128 matrix, 5-mm section thickness, and 16 transverse sections in the volume. A spatial saturation slab was implanted inferior to the acquired region to minimize the inflow effect from the carotid arteries. Before the administration of the contrast agent, the baseline longitudinal relaxation time (T1_0_) values were calculated from images acquired with different flip angles (4°, 8°, 15°, and 25°). The dynamic series involved the use of the same sequence with a 15° flip angle. After four acquisitions of the dynamic baseline scanning, a standard dose (0.1 mmol/kg body weight) of gadopentetate dimeglumine (Gd-DTPA; Magnevist, Bayer-Schering, Burgess Hill, UK) was administered by a power injector through a cannula placed in the antecubital vein at a rate of 3 mL/s and immediately followed by a saline flush. A total of 80 volumes were acquired with a temporal resolution of 3.3 s.

### Data Analysis

The ADC maps were reconstructed on a pixel-by-pixel basis using software integral to the MRI unit. The ADC was measured on ADC maps by drawing the region of interest (ROI) on the primary tumor at the level of its largest diameter by a radiologist with over 20 years of experience in head and neck imaging with the aid of the T2-weighted MR images and the T1-weighted post-contrast MR images. Visually large cystic or necrotic areas were avoided.

For DCE-PWI analysis, all of the data were processed in MATLAB 7.0 (The Mathworks, Natick, MA, USA). The signal intensities of the DCE-PWI data were converted from the contrast agent concentration by solving the nonlinear relationship between the signal intensity and the contrast agent concentration [29]. The extended Kety model [11] was adopted for the pharmacokinetic analysis in a voxel-wise manner. The arterial input function was extracted using the blind source separation algorithm [30], in which the extracted arterial voxel was located on the adjacent carotid artery of each patient. ROIs were manually drawn in the primary tumor on DCE MR images by the radiologist in a manner similar to that described for the DWI analysis. The following pharmacokinetic parameters were obtained: the volume transfer rate constant (*K* ^*trans*^), volume fraction of the extravascular extracellular space (*V*_*e*_), plasma volume (*V*_*p*_), and redistribution rate constant (*K*_*ep*_) which equals the ratio of *K* ^*trans*^ to *V*_*e*_.

The T-stage was recorded according to the 2010 cancer staging system revised by the American Joint Committee on Cancer. The gross tumor volume (GTV) of the primary tumor was calculated with a CT-based three-dimensional radiation treatment planning system. The SUV and the MTV of the primary tumor were measured from attenuation-corrected FDG PET images using the PMOD software (PMOD Technologies Ltd, Zurich, Switzerland). The boundaries were drawn large enough to include the primary tumor. An SUV threshold of 2.5 was used to delineate the MTV [31,32]. The contour around the target lesion inside the boundaries was automatically produced and the voxels presenting SUV intensity > 2.5 within the contouring margin were incorporated to define MTV. The SUV, MTV, and TLG of the lesion were automatically demonstrated by the software. The TLG was calculated as the product of the lesion mean SUV and the MTV.

### Outcome Determination and Statistical Analysis

Local control was measured from the first day of treatment to the time of local failure or the last follow-up. Local failure was determined by histological confirmation (biopsy or surgical resection) or by a serial increase in lesion size on the follow-up imaging for at least 1 year. We used logistic regression analyses to analyze the relationship between the 2-year local control and the following baseline variables: the pretreatment Hb level, T-stage, tumor location, GTV of the primary tumor, SUV_max_, MTV, and TLG on FDG-PET/CT, ADC on DWI, and *K* ^*trans*^, V_e_, *V*_*p*_ and *K*_ep_ on DCE-PWI. We used the median values of the variables as the cutoff levels for the analysis [4,25]. The local control rates were plotted using the Kaplan-Meier method and compared using the log-rank test. OHSCC patients were also divided into a local control and a local failure group. The same variables mentioned above were compared between these two groups using an independent Student’s *t*-test or the Pearson’s chi-square test. For all the analyses, *P* values < 0.05 (two-tailed) were considered statistically significant. The variables showing significant differences were subjected to multivariable logistic regression analysis. A receiver operating characteristic (ROC) curve analysis was then performed to determine the value that best predicted local failure. All statistical analyses were performed using SPSS software version 13.0 (SPSS Inc., Chicago, IL, USA).

## Results

Between August, 2010 and March, 2012, a total of 78 OHSCC patients underwent pretreatment DWI and DCE-PWI. Twenty patients were excluded from the analysis, 7 of whom had small or unevaluable (too necrotic) lesions, 9 had considerable artifact on DWI or PWI, and 4 were dead before the definite diagnosis of recurrence could be determined. Therefore, 58 patients were available for the analysis (29 oropharynx, 29 hypopharynx; 4 females and 54 males; median age, 48.5 ± 9.7 years). The patient characteristics are shown in Table 1. The pretreatment *K* ^*trans*^ of the primary lesion ranged from 0.22 to 1.43 min^-1^ (median, 0.62 min^-1^), *V*_*e*_ ranged from 0.03 to 0.83 (median, 0.20), and *V*_P_ ranged from 0.001 to 0.94 (median, 0.01). The ADC of the primary lesion ranged from 0.67 to 1.59 × 10^-3^ mm^2^/s (median, 0.96 × 10^-3^ mm^2^/s). After chemoradiation, the response rates in our sample of 58 patients were as follows: complete response (n = 46; 79.3%), partial response (n = 10; 17.2%), and stationary disease (n = 2; 3.4%). Univariate logistic analysis showed that tumor site (*P* =0.018), *K* ^trans^ (*P* =0.001), and *V*_*e*_ (*P* =0.028) were significantly associated with complete response.

**Table 1:** Baseline characteristics of OHSCC patients (n = 58).

**Characteristic**	
**Age (years)**	
median	48.5
range	34-78
**Sex (n (%) of patients)**	
Male	54 (93.1)
Female	4 (6.9)
**Hb value (g/dL)**	
median	14.4
range	6.1-18.3
**Tumor site (n (%) of patients)**	
Hypopharynx	29 (50)
Oropharynx	29 (50)
**Tumor volume (cm^3^)**	
mean	35.84
range	1.9-144
**T stage (n (%) of patients)**	
1	4 (6.9)
2	11 (19)
3	6 (10.3)
4	37 (63.8)
**N stage (n (%) of patients)**	
0	10 (17.2)
1	4 (6.9)
2	33 (56.9)
3	11 (19)
**Overall stage (n (%) of patients)**	
II	3 (5.2)
III	3 (5.2)
IVA	38 (65.5)
IVB	14 (24.1)

**Figure 1:**
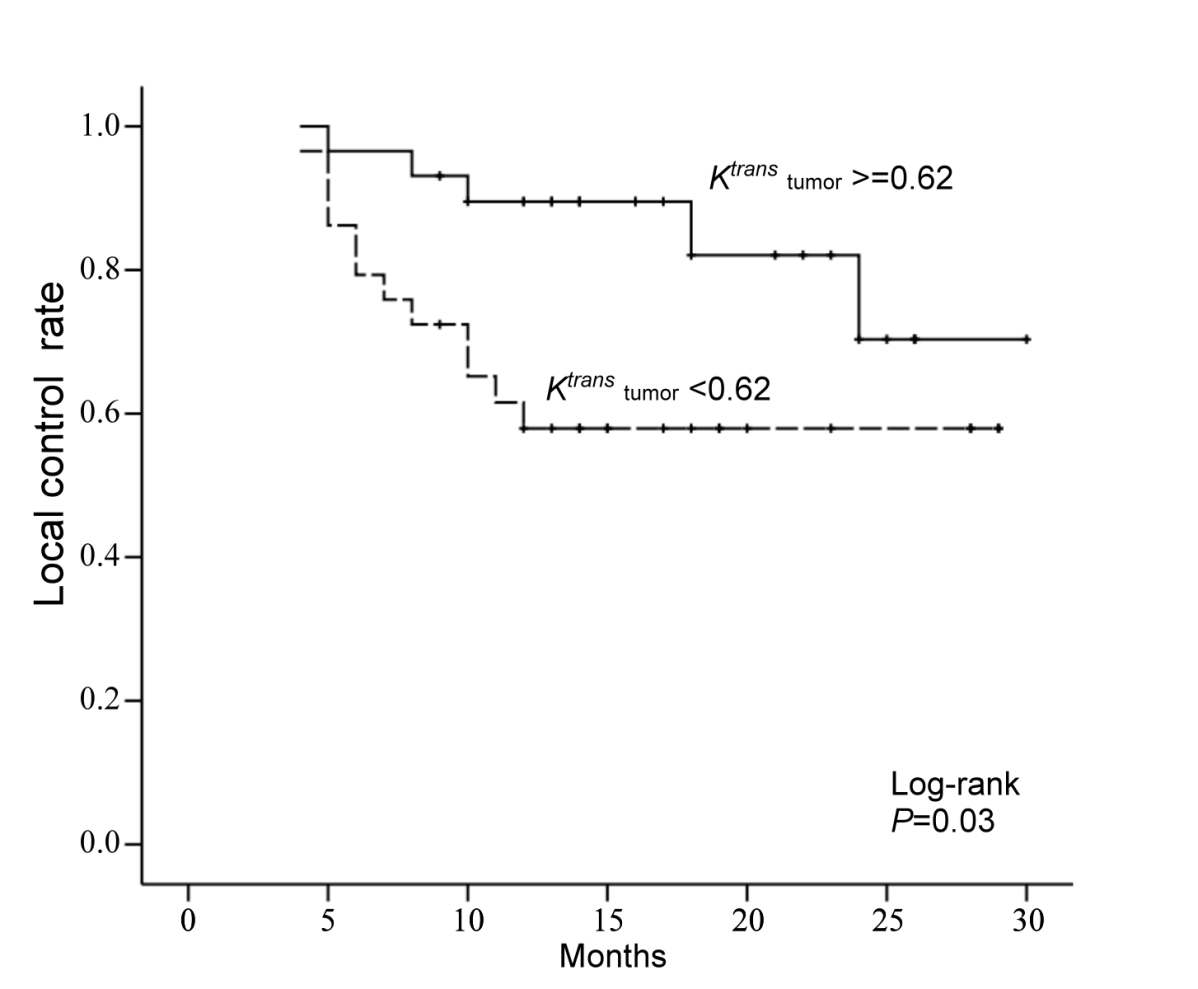
Kaplan-Meier analysis of local control rate stratified according to the median value of *K* ^*trans*^.

**Figure 2:**
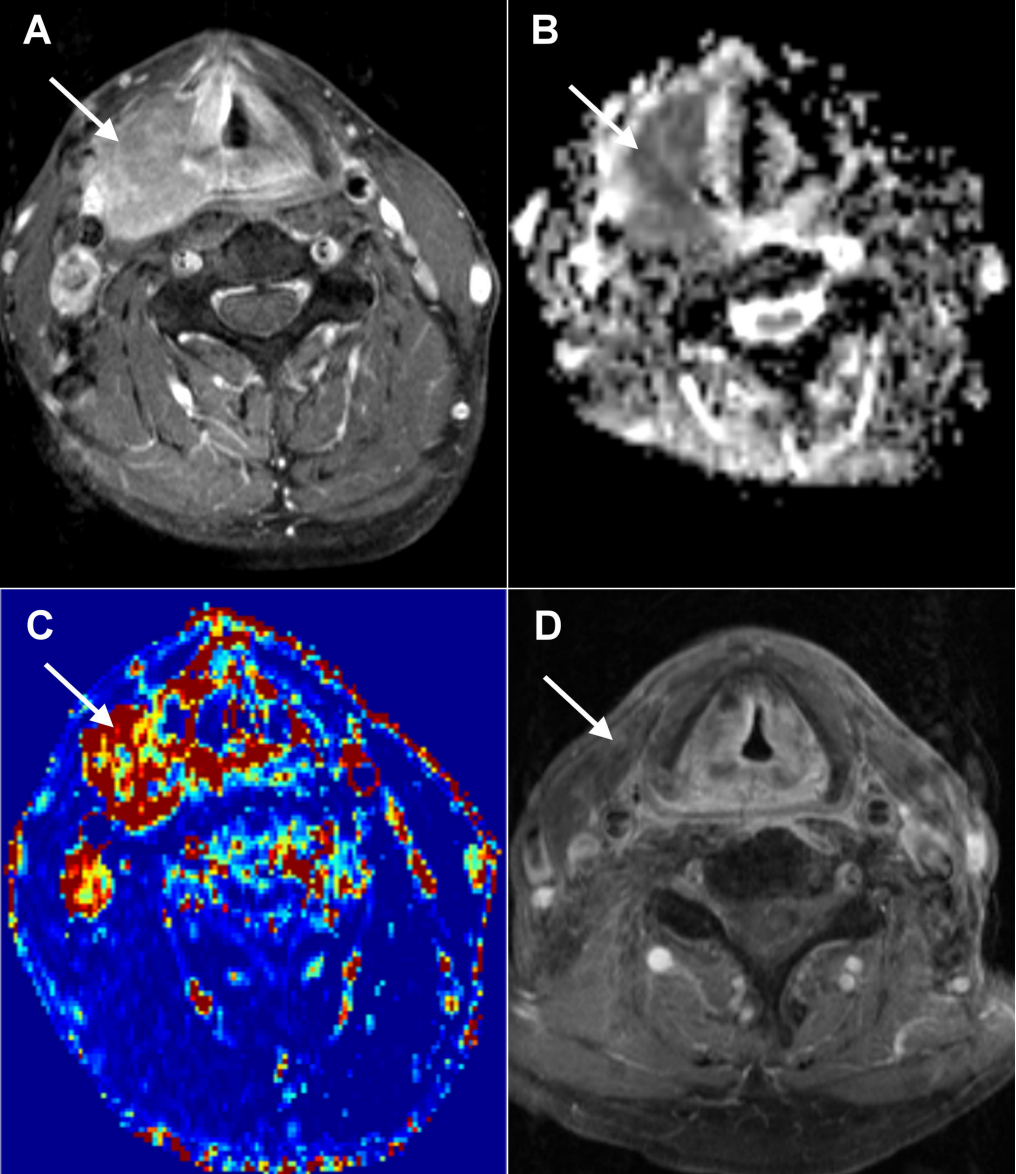
Local control in a 52-year-old man with hypopharyngeal SCC after chemoradiation. A. A pretreatment axial-enhanced MRI shows the right hypopharynx tumor (arrow). B. The corresponding PWI map shows the tumor with a *K* ^*trans*^ value of 0.614 min^-1^. C. The corresponding ADC map shows the tumor with an ADC value of 0.98 × 10^-3^ mm^2^/s. D. Post-treatment axial-enhanced MRI shows the regression of the right hypopharynx tumor. A 18-month clinical and imaging follow-up did not disclose any tumor growth.

**Figure 3:**
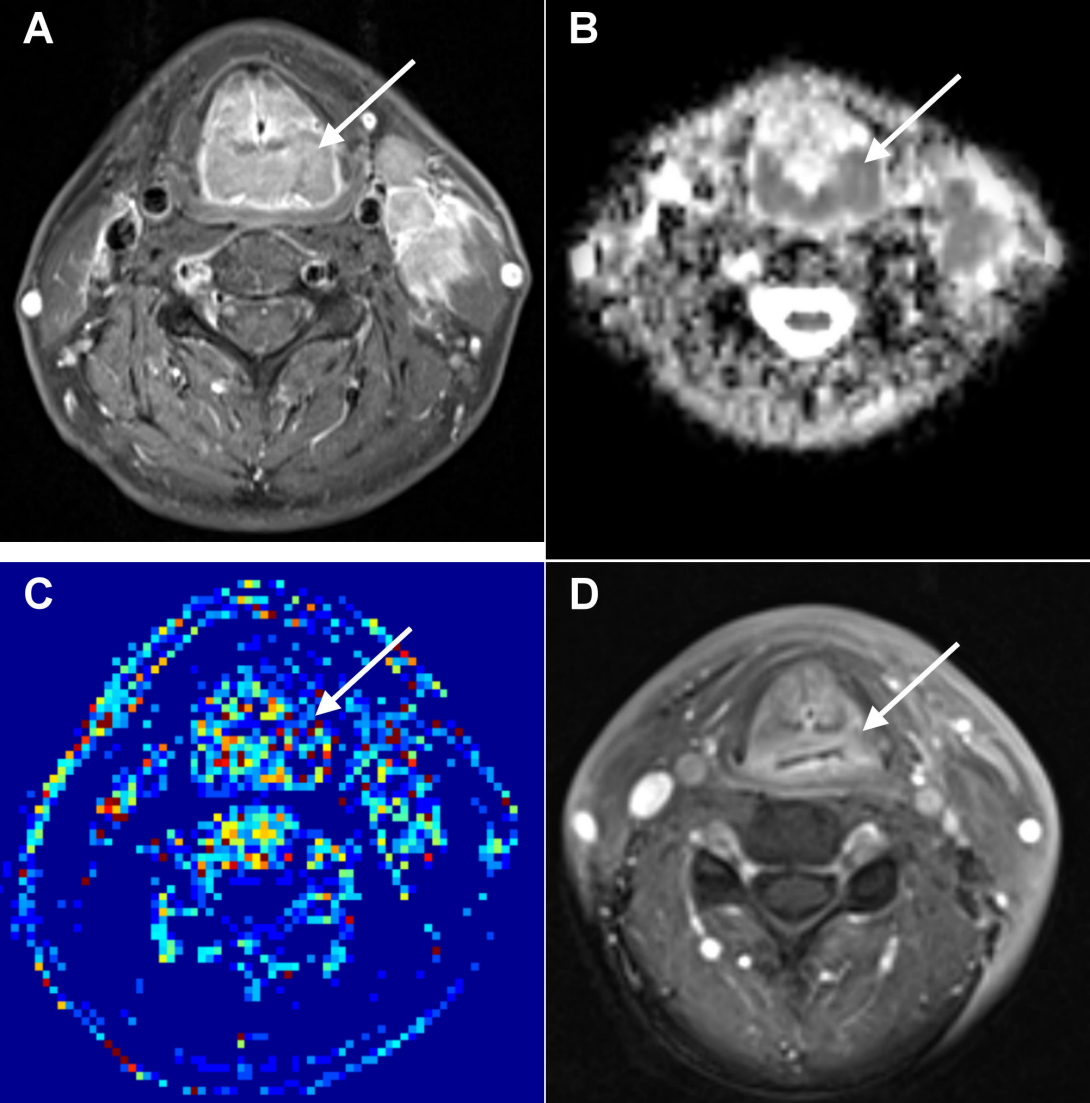
Local failure in a 61-year-old man with oropharyngeal SCC after chemoradiation. A. A pretreatment axial-enhanced MRI shows the oropharynx tumor (arrow). B. The corresponding PWI map shows the tumor with a *K* ^*trans*^ value of 0.336 min^-1^. C. The corresponding ADC map shows the tumor with an ADC value of 0.76 × 10^-3^ mm^2^/s. D. Post-treatment axial-enhanced MRI shows a residual left oropharynx tumor (arrow) which was proven by endoscopic biopsy.

After a median follow-up time of 19.2 months (range, 9–32.3 months), 41 (70.7%) of the 58 patients achieved local control, whereas 17 (29.3%) patients had local failure. Local failure cases were confirmed by pathology for the presence of a viable tumor in 11 patients, or by disease progression in the remaining six. In univariate analysis, patients with a high pretreatment *K* ^*trans*^ value had a significantly higher 2-year local control rate than those with a low *K* ^*trans*^ value (*P* = 0. 03, hazard ratio = 0.34, Figures 1-3). The other DCE-MRI parameters, including *V*_*e*_, *V*_P_ and *K*_*ep*,_ showed no association with local control. A tendency of a higher local control rate was found in patients with a low SUV_max_ as compared those with a high SUV_max_ (*P* = 0.06) and also in patients with oropharynx tumor as compared with those with hypopharyngeal tumor (*P* = 0.07). Patients with a low Hb level, high T-stage, high GTV, high MTV, or high TLG had lower 2-year local control rates than their counterparts, but the differences were not statistically significant. Patients with a high ADC level had similar local control rates as those with a low ADC value (*P* = 0.51; Table 2).

**Table 2:** Relationship between local failure and the variables.

	**n**	**2-year control**	P-value*	**Hazard Ratio**
**Hb (g/dl)**				
<12	12	50.0%	0.71	
> = 12	46	66.0%		
**Tumor site**				
Hypopharynx	29	53.5%	0.07	
Oropharynx	29	81.8%		
**T stage**				
T1-T2	15	75.0%	0.28	
T3-T4	43	59.5%		
**GTV (cm^3^)**				
<24.9	29	72.7%	0.25	
> = 24.9	29	51.5%		
**SUV**				
<15.31	28	78.2%	0.06	
> = 15.31	29	49.0%		
**MTV(cm^3^)**				
<23.28	28	74.9%	0.20	
> = 23.28	29	54.9%		
**TLG**				
<121.86	28	75.4%	0.18	
> = 121.86	29	51.5%		
***K*^trans^ (min^-1^)**				
<0.62	29	57.9%	0.03	0.34
> = 0.62	29	70.3%		
***V*_*p*_×10^3^**				
<0.01	33	66.1%	0.47	
> = 0.01	25	59.7%		
***V*_*e*_**				
<0.2	29	60.9%	0.14	
> = 0.2	29	68.2%		
***K*_*ep*_(min^-1^)**				
<2.63	29	61.1%	0.29	
> = 2.63	29	65.4%		
**ADC(×10^-3^mm^2^/s)**				
<0.96	27	64.8%	0.51	
> = 0.96	31	63.8%		

Abbreviations: GTV = Gross tumor volume; SUV = Standardized uptake value; MTV = Metabolic tumor volume; TLG = Total lesion glycolysis; ADC = Apparent diffusion coefficient*Log Rank test

**Figure 4:**
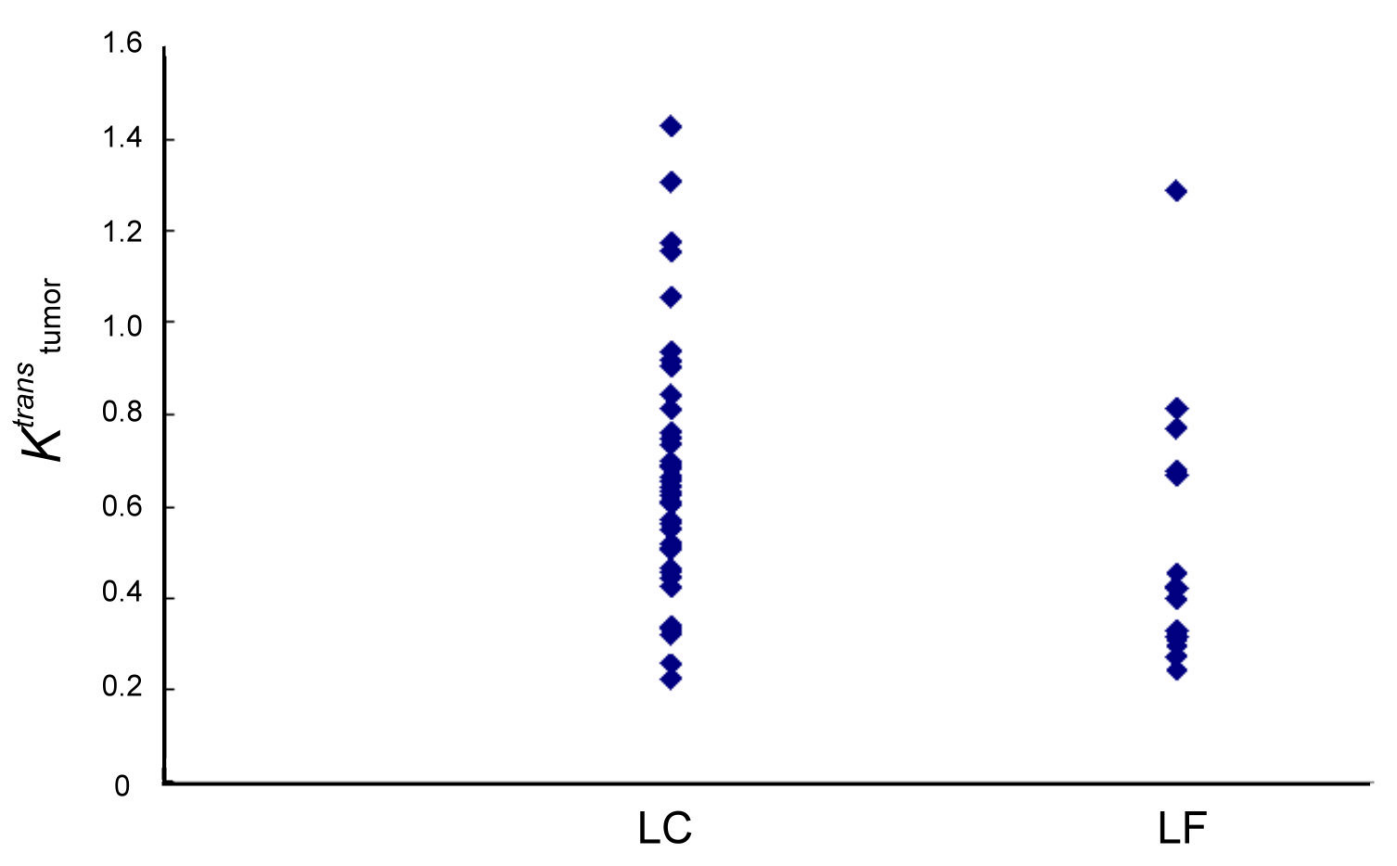
Scatterplots of *K* ^*trans*^ in the local control (LC, *n*= 41) and local failure (LR, *n=17*) groups.

**Figure 5:**
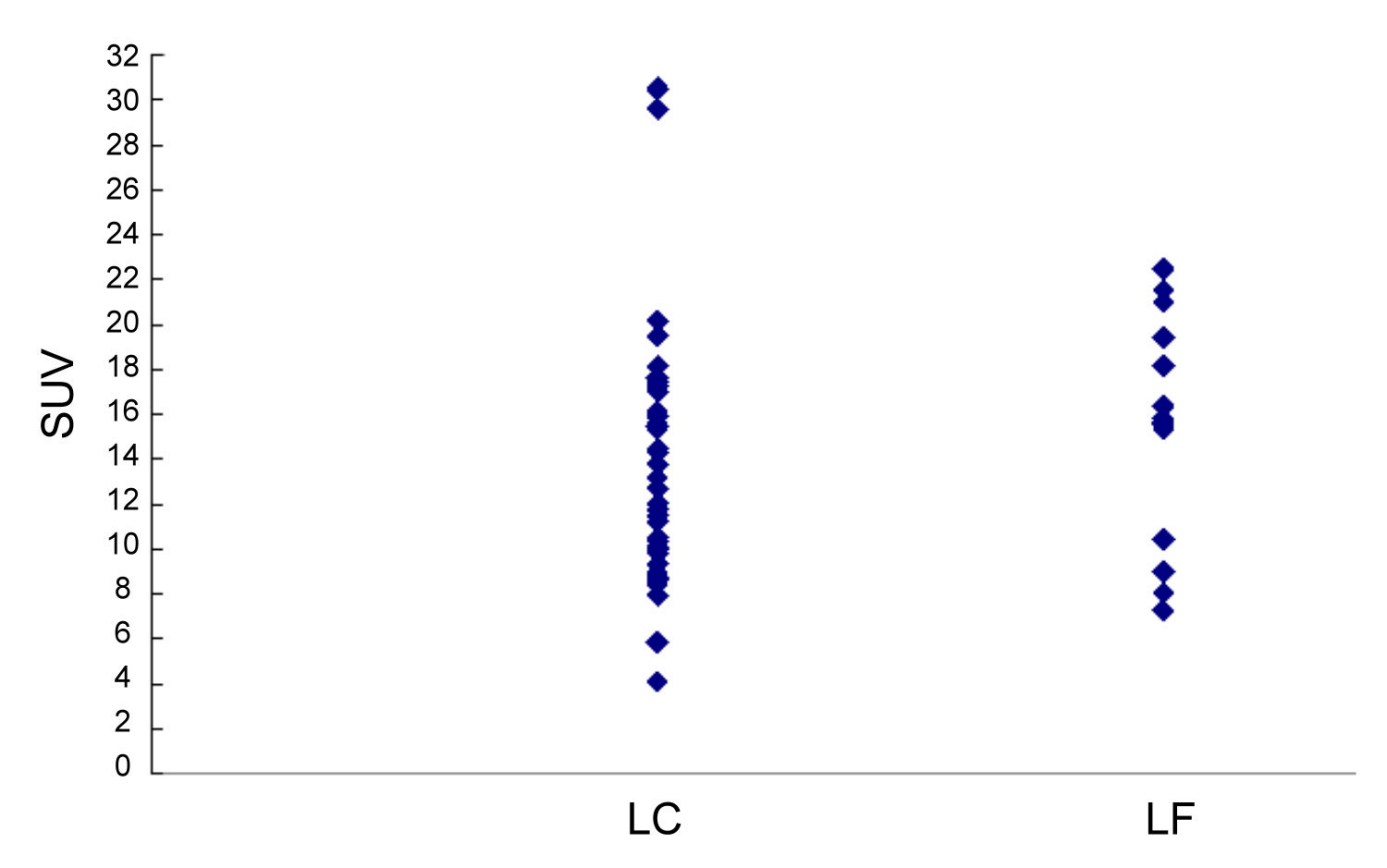
Scatterplots of SUV_max_ in the local control (LC, *n*= 41) and local failure (LR, *n=17*) groups.

**Figure 6:**
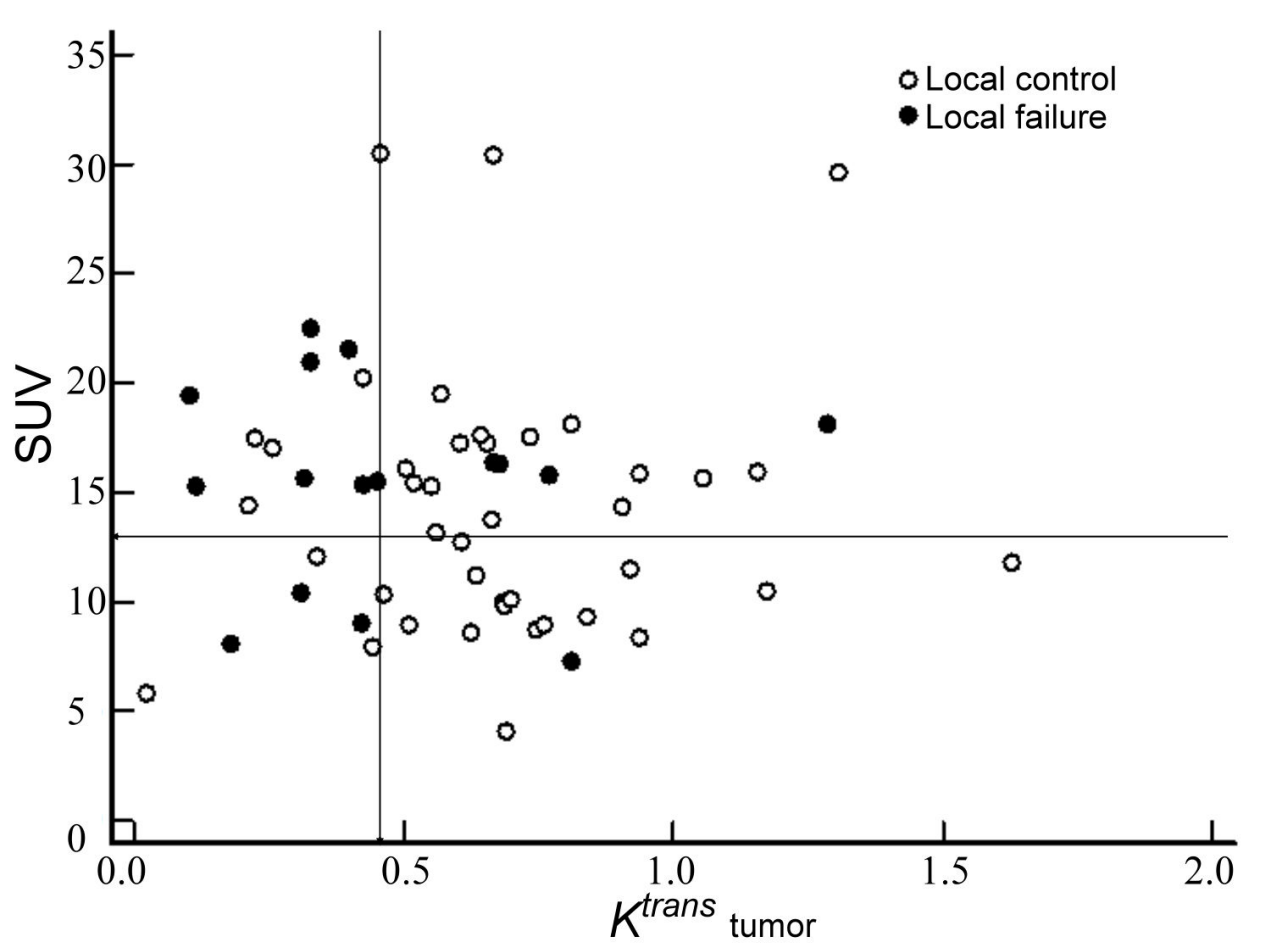
Scatterplots of the incidence of local failure with respect to the relationship between *K* ^*trans*^ and SUV_max_. The entire area of interest was divided into subsections (lines) using a cut-off of 0.45 min^-1^ for *K* ^*trans*^ and a cut-off of 13.76 g/mL for SUV _max_.

**Figure 7:**
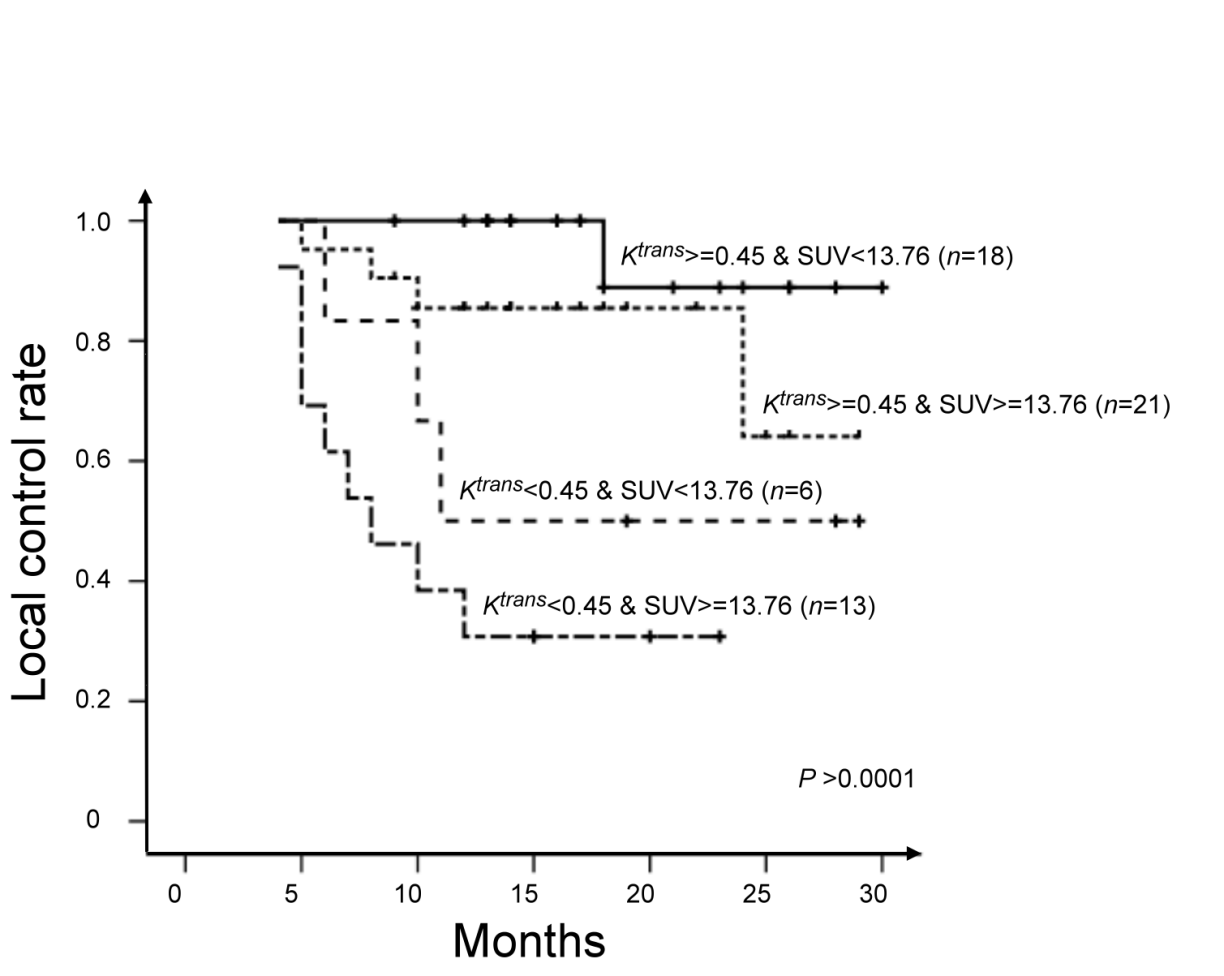
Subgroup analysis of local control rates according to different combinations of *K* ^trans^ (cut-off = 0.45 min^-1^) and SUV_max_ (cut-off = 13.76 g/mL).

When the local control and local failure groups were compared, the average value of *K* ^*trans*^ for the local control group was significantly higher than that of the local failure group (0.68 min^-1^ vs. 0.45 min^-1^, *P* = 0.001, independent Student’s *t*-test; Figure 4). The value of SUV_max_ for the local control group was also higher than that of the local failure group, but without statistical significance (Figure 5). The oropharynx tumors were associated with a significantly higher local control rate than the hypopharynx tumors (82.7% vs. 58.6%, *P* = 0.04, chi-square test). No significant difference was found for the ADC or the other variables between both groups (Table 3). The multivariable logistic regression demonstrated that *K* ^*trans*^ was the only significant predicator (*P* = 0.04) of local control. A *K* ^*trans*^ of 0.453 min^-1^, determined by the ROC curve as a cutoff value for predicting local control, attained 70.6% sensitivity, 82.9% specificity, 63.1% positive predictive value and 87.2% negative predictive value. Because SUV_max_ value was marginally associated with local control (*P* = 0.06), we further assessed whether the SUV_max_ could improve the predictive value of *K* ^*trans*^ for predicting local control. ROC analysis identified a SUV_max_ of 13.76 g/mL as the optimal cutoff for predicting local control. By combining the two variables, we were able to divide the patients into four distinct local control groups: Group 1, K^*trans*^ (min^-1^) ≥0.45 and SUV (g/mL) <13.76 (*n* = 18); *K* ^*trans*^ (min^-1^) ≥0.45 and SUV (g/mL) ≥13.76 (*n* = 21); *K* ^*trans*^ (min^-1^) <0.45 and SUV (g/mL) <13.76 (*n* = 6); *K* ^*trans*^ (min^-1^) <0.45 and SUV (g/mL) ≥13.76 (*n* = 13). The local failure rates of Groups 1, 2, 3, and 4 were 5.6% (1/18), 19.0% (4/21), 50.0% (3/6), and 69.2% (9/13), respectively (Figure 6). The four groups were found to differ significantly in terms of 2-year local control rates (88.9%, 64.1%, 50.5%, and 30.8%; *P* < 0.0001, Figure 7)

**Table 3:** Comparison of variables between the local control and local failure groups.

	**Local control**	**Local failure**	***P* value**	**Odds ratio**
**Hb (g/dL)**	14±2.1	13.7±2.8	0.69*	
**Tumor site**				
Hypopharynx	17	12	0.04^†^	0.30
Oropharynx	24	5		
**T stage**				
T1-T2	12	3	0.51^†^	
T3-T4	29	14		
**GTV (mL)**	36±31.9	35.5±23.5	0.96*	
**SUV (g/mL)**	14.2±6	15.5±4.7	0.45*	
**MTV (mL)**	27.2±24.6	27.2±16.2	0.99*	
**TLG (g)**	168.4±173.8	171.3±116	0.95*	
**K^trans^ (min^-1^)**	0.7±0.3	0.5±0.3	0.01*	0.06
***V*_*p*_×10^3^**	0.04±0.1	0.13±0.3	0.20*	
***V*_*e*_**	0.3±0.2	0.2±0.1	0.10*	
***K*_*ep*_ (min^-1^)**	4.2±3.4	3.2±2.2	0.30*	
**ADC(×10^-3^mm^2^/s)**	1±0.2	1±0.1	0.77*	

Abbreviations: GTV = gross tumor volume; SUV = standardized uptake value; MTV = metabolic tumor volume; TLG = total lesion glycolysis; ADC = apparent diffusion coefficient*Independent Student’s *t*-test ^†^Pearson’s chi-square test

## Discussion

In this study, we evaluated the capability of pharmacokinetic parameters derived from pretreatment DCE-PWI and DWI in combination with other potential factors to predict the local control of cancer after chemoradiation in patients with OHSCC. This study showed that only the pretreatment *K* ^*trans*^ of the primary tumor was significantly associated with local control rate, and *K* ^*trans*^ was also significantly higher in the local control group than in the local failure group. Since *K* ^*trans*^ is the pharmacokinetic parameter of DCE-PWI that reflects tumor vascularity and permeability, it can influence the delivery of chemotherapy drugs as well as oxygen during radiotherapy. Accordingly, it is expected that tumors with a high *K* ^*trans*^ would be associated with good treatment response. This explanation is consistent with the results of the present study. A better therapeutic response in lesions with high *K* ^*trans*^ values has been documented in cervical cancer treated with radiotherapy [33] and colorectal cancer treated with chemoradiation [34]. However, another DCE-MRI study of colorectal liver metastases undergoing 5-fluorouracil treatment reported that the pretreatment *K* ^*trans*^ did not predict the treatment response [35]. Therefore, the predictive value of *K* ^*trans*^ for the treatment response may vary among different therapeutic strategies and tumor types. A few studies have investigated the capability of pretreatment DCE-PWI to predict the response of HNSCC to chemoradiation [7,16,18]. Ceo et al. [16] reported that an increase in blood volume in the primary tumor during radiotherapy was associated with local control while the pretreatment blood volume and blood flow were not significant predictors of the outcome. In a series of 33 patients with HNSCC, Kim et al. [7] demonstrated that the pretreatment *K* ^*trans*^ of metastatic nodes of complete response patients was significantly higher than that observed for the partial response patients; no difference was found in *V*_*e*_ between the two patient groups. In a recent study of 15 HNSCC patients, Jansen et al. [18] showed that the standard deviation of *K* ^*trans*^ of metastatic nodes was a significant predictor of the short-term response, and again, *V*_*e*_ and *K*_*ep*_ were not of predictive value. Our study first documented that the *K* ^*trans*^ of the primary tumor was the only DCE-PWI parameter associated with local control in OHSCC treated with chemoradiation, whereas *V*_*e*_, *V*_*p*_ and *K*_*ep*_ did not function as predictors. A cutoff value of 0.4528 minutes^-1^ for *K* ^*trans*^ attained 70.6% sensitivity and 82.9% specificity in predicting local control.

With regard to the usefulness of the pretreatment ADC for predicting the response to chemoradiation in HNSCC, one study demonstrated a significantly lower pretreatment ADC in lymph node metastases in complete responders than in partial responders [15], and two other reports found a significant correlation between the pretreatment ADC of a primary lesion and local treatment failure [14,17]. However, a conflicting result was reported in a larger study of 50 HNSCC patients [13]. The authors found that the pretreatment ADC of a primary tumor did not predict the chemoradiation response. The lack of an association between local control and the pretreatment ADC value of primary tumors in OHSCC patients observed in our study is consistent with the results of King et al. [13] and suggests that the primary tumor cellularity may not be a significant predictor of chemoradiation success.

SUV reflects the glucose metabolism of a tumor and is the widely used PET parameter. Pretreatment SUV_max_ of FDG PET has been reported to be an independent prognostic factor of local control in HNSCC treated by radiotherapy or chemotherapy [25–27]. However, some other studies have reported the opposite result [31,36–40] ; instead of SUV_max_, MTV and TLG have been suggested to be adverse prognostic factors for local failure [31,38,39]. In the present study, we found that patients with high values of SUV_max_, MTV or TLG had lower local control rates than those patients with lower values of these parameters, but only the SUV_max_ value was marginally significantly associated with 2-year local control (*P* = 0.06). Possible explanations for the discrepancies between the results of previous studies and the present study pertain to the variability in quantitative PET measurements that depend on technical parameters of data acquisition and processing, including the segmentation algorithm and the threshold chosen for these parameters. A larger-sized series with standardization of the quantification techniques of SUV, MTV, and TLG is needed to clarify their predictive values. Nevertheless, our study have showed that the combination of the low *K* ^*trans*^ and high SUV_max_ of the primary tumor signified a subgroup of OPSCC patients at high risk of local failure. Consequently, these subjects are candidates for a more aggressive treatment approach.

Our findings agree with previous studies that patients with hypopharyngeal SCC had a worse outcome than patients with oropharyngeal SCC [24,32,39,41]. In this study, the tumor location (oropharynx versus hypopharynx) was weakly associated with the 2-year local control rate and showed slightly significant difference between the control and failure group, in which oropharyngeal SCC had better chemoradiation response than hypopharyngeal SCC. Therefore, oropharyngeal SCC showed a trend towards more local control than hypopharyngeal SCC, although the multivariable analysis revealed that the difference was not statistically significant. We suggest that more caution should be paid in chemoradiation selection and surveillance for hypopharyngeal SCC than for oropharyngeal SCC.

Moreover, treatment response in HNSCC patients after radiotherapy has been reported to be significantly associated with the pretreatment hemoglobin levels and T stage [14,19,20,22,25–27],, although there are some conflicting reports. Strongin et al [24] reported that the GTV was the best predictor of tumor control of HNSCC and found that the T stage was not a significant factor. Ohnishi et al [4] demonstrated that the GTV was more predictive of local control than the pretreatment Hb level or T-stage. However, according to Nathu et al. [22], the impact of the GTV was less pronounced than that of the T-stage on the local control of oropharyngeal SCC treated with radiotherapy. Moreover, variability in volume calculations of the GTV between observers is problematic. Our results support those studies that reported a lack of predictive power of the Hb level, T stage, and GTV, and suggest that these factors cannot be used as predictors for local control of OHSCC treated with chemoradiation.

## Conclusions

Our results demonstrated that the pretreatment *K* ^*trans*^ of the primary tumor was a significant predictor of local failure of OHSCC treated with chemoradiation, implying that tumor vascularity and permeability are more predictive of local control than tumor cellularity, metabolism, volume, and T-stage. The combination of *K* ^*trans*^ with SUV_max_, an imaging parameter that was marginally associated with local control rate in our study, improved the prognostic stratification of OHSCC patients who received chemoradiation with curative intent.
